# Enhancing reporting quality and impact of early phase dose-finding clinical trials: CONSORT Dose-finding Extension (CONSORT-DEFINE) guidance

**DOI:** 10.1136/bmj-2023-076387

**Published:** 2023-10-20

**Authors:** Christina Yap, Olga Solovyeva, Johann de Bono, Jan Rekowski, Dhrusti Patel, Thomas Jaki, Adrian Mander, Thomas R Jeffry Evans, Richard Peck, Kathryn S Hayward, Sally Hopewell, Moreno Ursino, Khadija Rerhou Rantell, Melanie Calvert, Shing Lee, Andrew Kightley, Deborah Ashby, An-Wen Chan, Elizabeth Garrett-Mayer, John D Isaacs, Robert Golub, Olga Kholmanskikh, Dawn Richards, Oliver Boix, James Matcham, Lesley Seymour, S Percy Ivy, Lynley V Marshall, Antoine Hommais, Rong Liu, Yoshiya Tanaka, Jordan Berlin, Aude Espinasse, Munyaradzi Dimairo, Christopher J Weir

**Affiliations:** 1 Institute of Cancer Research, London SM2 5NG, UK; 2 Royal Marsden NHS Foundation Trust, London, UK; 3 MRC Biostatistics Unit, Cambridge University, Cambridge, UK; 4 Computational Statistics Group, University of Regensburg, Regensburg, Germany; 5 Centre For Trials Research, Cardiff University, Heath Park, Cardiff, UK; 6 Institute of Cancer Sciences, CR-UK Beatson Institute, University of Glasgow, Glasgow, UK; 7 Department of Pharmacology and Therapeutics, University of Liverpool, Liverpool, UK; 8 Hoffmann-La Roche, Basel, Switzerland; 9 Departments of Physiotherapy, and Medicine (Royal Melbourne Hospital), University of Melbourne, VIC, Australia; 10 Florey Institute of Neuroscience and Mental Health, University of Melbourne, Melbourne, VIC, Australia; 11 Oxford Clinical Research Unit, NDORMS, University of Oxford, Oxford, UK; 12 ReCAP/F CRIN, INSERM, Paris, France; 13 Unit of Clinical Epidemiology, CHU Robert Debré, APHP, URC, INSERM CIC-EC 1426, Reims, France; 14 INSERM Centre de Recherche des Cordeliers, Sorbonne University, Paris Cité University, Paris, France; 15 Health data and model driven approaches for Knowledge Acquisition team, Centre Inria, Paris, France; 16 Medicines and Healthcare Products Regulatory Agency, London, UK; 17 Centre for Patient Reported Outcomes Research, Institute of Applied Health Research, University of Birmingham, Birmingham, UK; 18 Birmingham Health Partners Centre for Regulatory Science and Innovation, University of Birmingham, Birmingham, UK; 19 National Institute for Health and Care Research (NIHR) Applied Research Collaboration West Midlands, University of Birmingham, Birmingham, UK; 20 NIHR Research Blood and Transplant Research Unit in Precision Transplant and Cellular Therapeutics, University of Birmingham, Edgbaston, Birmingham, UK; 21 NIHR Birmingham Biomedical Research Centre, Institute of Translational Medicine, University Hospital NHS Foundation Trust, Birmingham, UK; 22 Columbia University Mailman School of Public Health, New York, NY, USA; 23 Lichfield, UK; 24 School of Public Health, Imperial College London, London, UK; 25 Department of Medicine, Women’s College Research Institute, University of Toronto, Toronto, ON, Canada; 26 Center for Research and Analytics, American Society of Clinical Oncology, Alexandria, VA, USA; 27 Translational and Clinical Research Institute, Newcastle University, Newcastle upon Tyne, UK; 28 Musculoskeletal Unit, Newcastle upon Tyne Hospitals NHS Foundation Trust, Freeman Hospital, Newcastle upon Tyne, UK; 29 Department of Medicine, Northwestern University Feinberg School of Medicine, 633 Clark Street, Evanston, IL, USA; 30 Federal Agency for Medicines and Health Products, Brussels, Belgium; 31 European Medicines Agency, Amsterdam, Netherlands; 32 Clinical Trials Ontario, MaRS Centre, Toronto, ON, Canada; 33 Bayer, Berlin, Germany; 34 Strategic Consulting, Cytel (Australia), Perth, WA, Australia; 35 Investigational New Drug Programme, Canadian Cancer Trials Group, Cancer Research Institute, Queen’s University, Kingston, ON, Canada; 36 Investigational Drug Branch, Cancer Therapy Evaluation Program, Division of Cancer Treatment and Diagnosis, National Institute of Health, Bethesda, MD, USA; 37 Department of Clinical Research, National Cancer Institute, Boulogne-Billancourt, France; 38 Bristol Myers Squibb, New York, NY, USA; 39 First Department of Internal Medicine, University of Occupational and Environmental Health, Kitakyushu, Japan; 40 Vanderbilt-Ingram Cancer Center, Nashville, TN, USA; 41 Division of Population Health, Sheffield Centre for Health and Related Research, University of Sheffield, Sheffield, UK; 42 Edinburgh Clinical Trials Unit, Usher Institute, University of Edinburgh, Edinburgh, UK

## Abstract

The CONSORT (CONsolidated Standards Of Reporting Trials) 2010 statement is the standard guideline for reporting completed randomised trials. The CONSORT Dose-finding Extension (DEFINE) extends the guidance (with 21 new items and 19 modified items) to early phase dose-finding trials with interim dose escalation or de-escalation strategies. Such trials generally focus on safety, tolerability, activity, and recommending dosing and scheduling regimens for further clinical development. These trials are often inadequately reported, hampering their informativeness and making evidence informed decisions difficult. The CONSORT-DEFINE guidance aims to develop an international, consensus driven guideline for reporting early phase dose-finding trials to promote transparency, completeness, reproducibility, and facilitate the interpretation of the results. The CONSORT-DEFINE guidance provides recommendations for essential items that should be reported in early phase dose-finding trials to promote greater clarity, reproducibility, informativeness, and usefulness of results.

Early phase dose-finding (EPDF) or dose escalation or de-escalation trials, commonly known as phase 1 or phase 1 or 2 trials, are an integral part of clinical development. EPDF trials typically evaluate new interventions that can be given in different doses and can be pharmacological (chemical or biological—eg, drugs, vaccines, cell therapies, gene therapies), non-pharmacological (eg, radiotherapy, rehabilitation, devices, digital therapies), or a combination of both. These trials require interim decisions on dosing changes of an intervention and generate data on safety and other information such as pharmacokinetics, pharmacodynamics, biomarker, or clinical activity to guide dosing selection and future clinical development.^1^ ^2^ ^3^ ^4^ In this article, a broad definition of “dose” is applied, because terms such as “dose finding,” “dose escalation,” “dose de-escalation,” “dose expansion,” and “dose level” are widely used. Here, dose might refer not only to the amount but also to the frequency, intensity, or duration of an intervention.^5^ The term could therefore be regarded as synonymous and used interchangeably with dosage or dosing regimen, or unit dose, and it can apply to interventions given alone or in combination (see the glossary in box 1 for details).

Box 1 **Glossary**

**Activity**

A measure of the physiological response that an intervention produces.

**Algorithm based (rule based) design**

A trial design that uses a simple set of predefined algorithms or rules to guide the decision making process for dose escalation or de-escalation. Examples include traditional 3+3, accelerated titration, and pharmacologically guided dose escalation designs.^6^ ^7^

**Biomarker substudy**

A part of a clinical trial that investigates biomarkers, which are “a defined characteristic that is measured as an indicator of normal biological processes, pathogenic processes, or biological responses to an exposure or intervention, including therapeutic interventions. Biomarkers could include molecular, histological, radiographic, or physiological characteristics. A biomarker is not a measure of how an individual feels, functions, or survives.”^8^

**Clinical benefit**

A favourable effect on a meaningful aspect of how a participant feels, functions, or survives as a result of an intervention.^9^

**Delphi survey**

A series of questionnaires used sequentially to gather diverse opinions that allow experts to develop ideas about potential future developments around an issue. The questionnaires are developed throughout the process in relation to the responses given by participants.

**Dose**

In this article, dose is defined broadly and can be considered synonymous with dosage or dosing regimen (dose or schedule), or a unit dose. The unit dose is the amount or intensity of an intervention (eg, drug quantity, radiotherapy, exercise level), or the extent to which a participant might be exposed to an intervention on a single occasion. Information on dosage should include aspects of the intervention that describe how many times it was delivered and for how long—such as the number of sessions; their schedule; and their duration, intensity, or dose.^5^

**Dose escalation or de-escalation**

An incremental increase or decrease (or up-titration or down-titration) in the strength of any intervention (eg, a drug or exercise intensity level) to improve its tolerability or maximise its pharmacological or clinical effect.

**Dose limiting criteria**

Effects or markers that are presumably related to the intervention and that either are considered unacceptable or show the desired level of effect has been achieved and a further increase in dose is not required.^10^

**Dose limiting toxicity**

Side effects of an intervention that are serious enough to prevent an increase in the dose of that intervention.^7^

**Dosing regimen or dosage**

See dose.

**Early phase dose-finding trial**

An early phase trial where different doses of the investigated intervention are given to groups of participants, with interim assessments of the safety/tolerability (and other markers such as activity) of the intervention.

**Estimand framework**

Estimands provide a structural framework to define the target of estimation for a particular clinical trial objective.^11^ ^12^ They require to specify the treatment condition of interest, the population targeted by the clinical question, the variable of interest or endpoint used to answer that question, the handling strategies for intercurrent events (ie, events occurring after treatment initiation that affect either the interpretation or the existence of the measurements associated with the clinical question), and a population level summary of the variable or endpoint.

**Expansion cohort or dose expansion**

A part of a dose escalation clinical trial that aims to accrue additional participants after an initial dose escalation part with different or targeted eligibility criteria to collect additional information on safety or activity.^13^

**Group**

Can refer to an intervention group or arm, or specifically defined subgroups of the targeted participant population based on, for example, participant or disease characteristics.

**Harms**

The totality of possible adverse consequences of an intervention or treatment; they are the direct opposite of benefits, against which they must be compared.^14^ Harms can comprise of adverse events, adverse (drug) reactions, toxicities, treatment emergent adverse events, or those that are intolerable by participants.^14^ ^15^ They can also include tolerability assessment using patient reported outcomes as complementary to investigators’ reporting.^16^ ^17^

**Interim analysis or review**

A statistical analysis or review of accumulating data from an ongoing trial (interim data) to inform trial adaptations (before the final analysis), which might or might not involve treatment group comparisons.^18^

**Model assisted design**

A trial design that combines a clearly predetermined algorithm to guide the dose escalation or de-escalation as in rule based designs, and an underlying statistical model, as in model based designs.^19^ Examples include the modified toxicity probability interval design^20^ and the bayesian optimal interval design.^21^

**Model based design**

A trial design that assumes a relation between the dose of the intervention given to the participant and the likelihood of the participant experiencing an effect (such as toxicity or activity) and uses a parametric model to estimate that association. Examples include the continual reassessment method,^22^ escalation with overdose control,^23^ and the efficacy-toxicity trade-off based design.^24^

**Multiple ascending dose**

A trial design where a small number of participants (healthy volunteers or participants) receive several doses of an intervention over time to assess safety or tolerability and pharmacokinetic and pharmacodynamic profiles. Doses can remain the same or increase within a participant. The dose level is subsequently escalated for further participants according to the protocol, assuming that strict safety, effect, or pharmacokinetic criteria are met.

**Operating characteristics**

Characteristics that relate to the statistical behaviour or performance of the trial design in answering research questions. These might include the probability of correctly selecting the correct dose, statistical power, false positive error rate, bias in estimation of treatment effect, or probability of each adaptation taking place.^18^ ^25^

**Pharmacodynamics**

Described as what a drug does to the body; pharmacodynamics refer to how the drug works and how it affects the body.

**Pharmacokinetics**

Described as what the body does to a drug; pharmacokinetics refer to the movement of the drug into, through, and out of the body. It includes the analysis of chemical metabolism and the measurement or modelling of a substance from the moment that it is used up to the point when it is completely eliminated from the body.

**Prespecified decision making criteria**

Planned or prespecified rules to guide decisions, describing whether, how, and when the proposed trial adaptations will be used during the trial. The criteria involve prespecifying a set of actions guiding how decisions about implementing the trial adaptations are made given interim observed data (decision rules). They also involve prespecifying limits or parameters to trigger trial adaptations (decision boundaries), for example, stopping boundaries that relate to prespecified limits regarding decisions to stop the trial or any treatment arms early.

**Single ascending dose**

A trial design in which a small number of participants receive one dose of a therapeutic intervention at a given dose level to assess safety or tolerability and characterise the pharmacodynamics and pharmacokinetics of the intervention. Single ascending dose trials are often conducted in a small number of healthy volunteers, although some trials recruit participants with a disease of interest. The dose is subsequently escalated for further participants according to the protocol, assuming that strict safety, effect, or pharmacokinetic criteria are met.

**Transition points**

The points or parts in a clinical trial when the decision can be made to proceed to the next stage or phase, such as from dose escalation to dose expansion, from phase 1 to phase 2, or from a single ascending dose to multiple ascending dose.

**Trial (design) adaptations**

Prespecified changes or modifications (defined in advance) that can be made to various aspects of a trial while it is ongoing without undermining the trial’s validity and integrity.^26^ These prespecified modifications are driven by accruing interim data.^27^ Examples include adjusting the doses; changing the predetermined sample size; stopping the trial early for efficacy, futility, or safety; and switching the allocated treatment of participants owing to a lack of benefit or safety issues.^18^

Incomplete or unclear information on design, conduct, and analysis when reporting results of EPDF trials can hamper the assessment of their reliability and conclusions about safety and efficacy,^28^ ^29^ and undermine public confidence in research. Accurate evaluation of EPDF trial findings is crucial to prevent inadequate dose selection, which frequently results in subsequent failures in phase 2 and phase 3 trials, regulatory submission delays, additional post-marketing commitments, or dose changes after approval due to excessive toxicities or lack of efficacy.^4^ ^30^

The use of more efficient but more complex dose escalation or de-escalation designs, such as model assisted or model based designs,^6^ ^31^ has risen from 1.6% (20/1235) in 1991-2006^32^ to 8.6% (68/788) of trials published in 2014-19.^6^ Recent findings based on a small sample of trials published in May-August 2022 showed a substantial increase in the use of such designs, reaching a proportion of 25.7% (9/35).^33^ These designs require the specification of more study design features.^3^ ^34^ ^35^ To enable readers to make informed judgments regarding potential biases and the reliability of EPDF study findings, it is imperative to provide greater clarity that helps them comprehend the design, understand how dose decisions were made, and ensure procedures and findings can be reproduced.^29^ ^36^

Neither CONSORT (CONsolidated Standards Of Reporting Trials) 2010^37^ ^38^ nor its extensions fully cover the features of EPDF trials.^29^ The need for a CONSORT extension for EPDF trials was largely driven by the fact that such trials^1^ ^18^ ^39^ have frequent reviews or analyses of interim data to make dosing decisions and other trial adaptations, might not be randomised (eg, 99.2% of oncology trials and 25.1% of non-oncology trials are non-randomised^40^), and have requirements and statistical considerations that differ from later phase randomised trials (covered by CONSORT 2010^37^ and related extensions such as Adaptive designs CONSORT Extension^18^). Moreover, globally, there are more phase 1 trials (n=18 716) than phase 3 trials (n=10 451) registered on ClinicalTrials.gov, based on trials first posted between 2018 and 2022. The number of phase 1 trials is most likely an underestimate, as it is not mandatory to register or report these trials on ClinicalTrials.gov.^41^

Conference and journal abstracts of EPDF trials communicate important clinical development of a new intervention. Since many EPDF trials remain unpublished,^42^ it is even more vital that these abstracts are well reported to increase their informativeness, as critical decisions might often be based on them.^43^

A methodological review to assess the reporting quality of 476 EPDF trial results publications from 2011 to 2020^40^ uncovered clear evidence of insufficient and inconsistent reporting of many aspects, including applicable CONSORT 2010 items. For instance, the rationale for the starting dose and the specification of planned or maximum sample size were reported in less than 25% and 40% of EPDF trials, respectively. Furthermore, reporting quality in EPDF trials has generally not improved over time.^40^

The prevalence of EPDF trials, their direct influence on late stage clinical development, and the urgent need to improve their reporting quality, further highlight the importance of a tailored reporting guidance. The CONSORT Dose-finding Extension (DEFINE) study aimed to enhance the transparency, completeness, reproducibility, and interpretation of EPDF trial results by developing and disseminating an extension to the CONSORT 2010 statement that is specific to EPDF trials, investigating interventions across all disease areas.^2^ ^29^

**Summary points**

Early phase dose-finding clinical trials are essential for clinical development because they lay the groundwork for further development and guide subsequent trials
The CONsolidated Standards Of Reporting Trials (CONSORT) 2010 statement focused on randomised trials and the new CONSORT Dose-finding Extension (DEFINE) guideline has been extended to broaden its applicability to early phase dose-finding trials with interim dose escalation or de-escalation strategies
After an international consensus-driven guideline development process using the EQUATOR (Enhancing QUAlity and Transparency Of health Research) methodological framework, 40 items specific to early phase dose-finding were recommended for inclusion in clinical trial reports
Inclusion of these CONSORT-DEFINE items in clinical trial reports could enhance transparency, completeness, reproducibility of methods, and usefulness of results in early phase dose-finding trials

## Methods

The CONSORT-DEFINE extension was developed following the EQUATOR methodological framework for guideline development^44^; the DEFINE protocol^2^ details how the project was developed. The project was approved for sponsorship by the Institute of Cancer Research’s Committee for Clinical Research (CCR5460). The UK Health Research Authority confirmed that research ethics approval was not required. Informed consent was obtained from both the Delphi survey and consensus meeting participants. The Standard Protocol Items: Recommendations for Interventional Trials (SPIRIT)-DEFINE (protocol guidance for EPDF trials)^45^ was developed in parallel with CONSORT-DEFINE.

### Generation of candidate reporting items

A methodological review of published EPDF trials identified features and deficiencies in reporting to inform the initial generation of the candidate items for CONSORT-DEFINE,^29^ ^40^ based broadly on existing reporting guidelines or recommendations including CONSORT 2010,^37^ ^38^ SPIRIT 2013,^46^ Adaptive designs CONSORT Extension,^18^ a checklist proposal for phase 1 dose-finding cancer trials,^47^ as well as consultation with experts. Further candidate items were generated through the analysis of peer reviewed literature, unpublished or grey literature (eg, regulatory and industry advisory documents), citation tracking, and expert opinion.^48^

### International Delphi process

**Fig 1:**
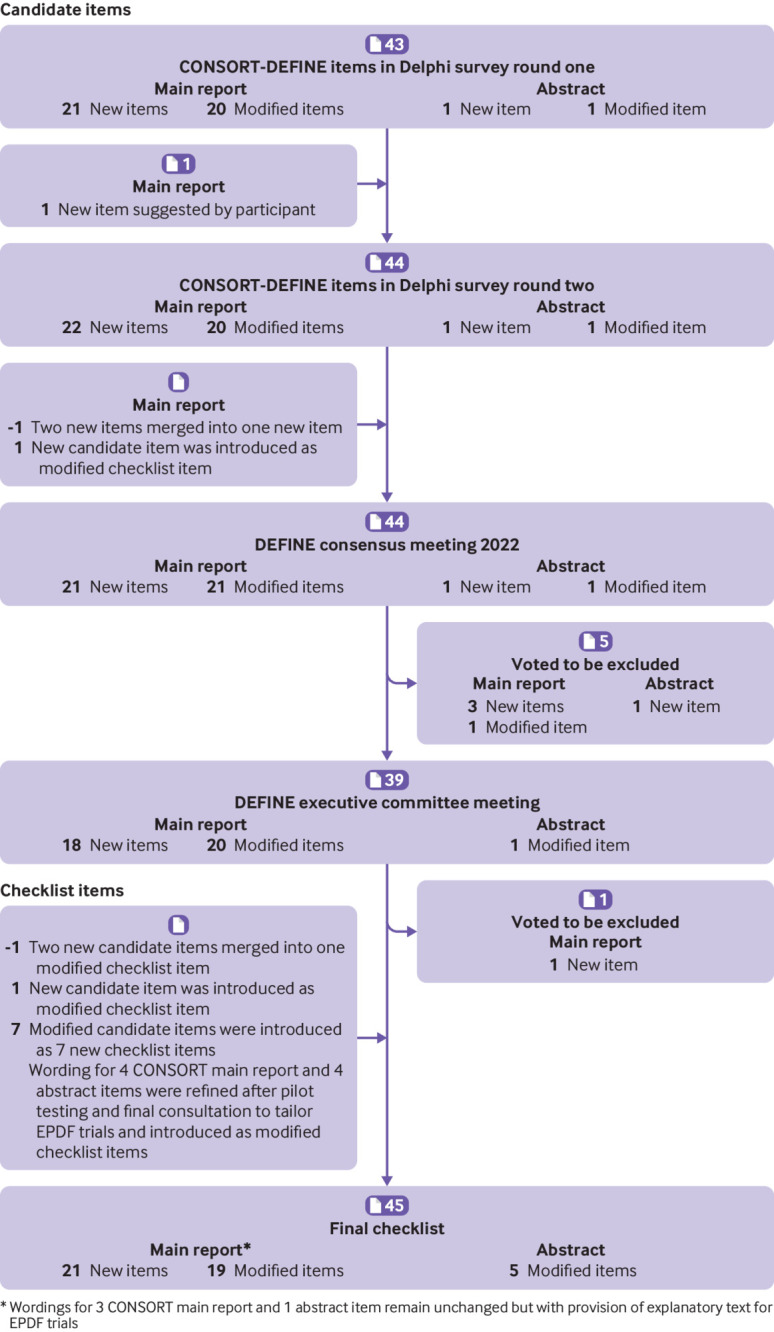
Development process of the CONSORT-DEFINE checklist items. CONSORT=CONsolidated Standards Of Reporting Trials; DEFINE=Dose-finding Extension; EPDF=early phase dose-finding

Through a Delphi survey (fig 1), the draft candidate items for the CONSORT-DEFINE checklist were presented for input and feedback from a large stakeholder group. The Delphi process was carried out following existing methodological guidance.^49^ ^50^ ^51^ Two hundred and six participants from 24 countries voted in round one (March-May 2022), and 151 participants voted in round two (May-June 2022) of the Delphi survey. Round two participants were shown the distribution of the item rating as well as their previous rating if they had completed round one.^48^

According to the prespecified decision criterion,^2^ items with at least 70% of respondents rating them as critically important were automatically included in the DEFINE checklist (fig S1 in web appendix 1). During the Delphi process, 34 of 44 candidate items considered over two rounds of the Delphi survey met the criteria for inclusion in the checklist, leaving 10 items for consideration at the consensus meeting (table S1 in web appendix 1). Further details, including the methods and results of the Delphi process and the qualitative and quantitative analyses, are reported within the DEFINE development process paper.^48^

### International consensus meeting

The online consensus meeting was held on 11-22 October 2022 and involved 32 international delegates from the academic, commercial, and regulatory sectors, and two patient and public involvement and engagement (PPIE) partners (tables S2 and S3 in web appendix 1). A series of slides was presented for each of the 10 candidate items: the Delphi voting results, alongside differences across stakeholder groups if they were present; supporting evidence of its importance; Delphi respondents’ comments; and examples of the item reported in scientific publications.

After discussion of each candidate item, delegates voted anonymously on whether to keep the item. Of 10 candidate items, two were recommended for inclusion in the CONSORT-DEFINE checklist (meeting the threshold of 70% of votes), five were rejected (receiving less than 50% of votes), and three (with 50-70% votes) were left for further deliberation by the DEFINE executive committee after the consensus meeting, of which two were recommended for inclusion in the checklist (fig S1 in web appendix 1).

### Final consultation and piloting of the checklist

Following the consensus meeting, participants and the DEFINE executive committee refined the wording of the checklist items and the corresponding explanatory text. The draft checklist was piloted six times using published and draft papers by international stakeholders (December 2022-February 2023) to evaluate its suitability and identify areas for improvement. The feedback gathered from the pilot testing further shaped the final version of the guideline, with the final wording agreed on by the DEFINE Executive Committee and consensus meeting participants.

## Results

Figure 1 presents the development journey of the CONSORT-DEFINE checklist items, from the Delphi survey to the consensus meeting, to the refinement of the checklist after final consultation and pilot testing. The final CONSORT-DEFINE guidance recommends that, in conjunction with the existing CONSORT 2010 items, 40 EPDF specific items (21 new and 19 modified) be included in EPDF trial reports. Table 1 presents the final CONSORT-DEFINE checklist for the main report of EPDF trials. It comprises the CONSORT 2010 checklist items and the recommended new or modified CONSORT-DEFINE items. The downloadable version of the CONSORT-DEFINE checklist for the main report is available in web appendix 2. Table 2 presents the CONSORT-DEFINE checklist for the abstract of EPDF trials.

**Table 1:** Recommended checklist items to consider in an early phase dose-finding clinical trial report from CONSORT 2010 and CONSORT-DEFINE checklists

Category and section	Standard CONSORT checklist item			CONSORT-DEFINE checklist item for EPDF Trials	
	Item No	CONSORT		Item No	CONSORT DEFINE
**Title and abstract**					
Title	1a	Identification as a randomised trial in the title		1a†	Identification as an early phase dose-finding (eg, first-in-human, dose escalation or de-escalation, phase 1, phase 1/2, expansion, dose titration) and, if applicable, randomised trial in the title or abstract
Abstract	1b	Structured summary of trial design, methods, results, and conclusions (for specific guidance, see CONSORT for abstracts)		1b	Structured summary of trial design, methods, results, and conclusions (for specific guidance, see CONSORT-DEFINE for abstracts)
**Introduction**					
Background and objectives	2a	Scientific background and explanation of rationale		2a.1†	Description of research question(s) and justification for undertaking the trial, including summary of relevant clinical studies (published and unpublished) examining benefits and harms for each intervention
				2a.2*	Summary of key findings from relevant non-clinical or preclinical research
				2a.3*	Summary of findings from previously generated preclinical and translational studies to support any planned biomarker substudies (where applicable)
	2b	Specific objectives or hypotheses		2b†	Specific objectives (eg, relating to safety, activity, pharmacokinetics, pharmacodynamics, recommended dose(s))
**Methods**					
Trial design	3a	Description of trial design (such as parallel, factorial) including allocation ratio		3a.1†	Description of trial design elements, such as dose escalation or de-escalation strategy, number of treatment groups, allocation ratio if relevant, and details of any prespecified trial adaptations
				3a.2*	Trial design schema to show the flow of major transition points (eg, dose escalation to dose expansion, phase 1 to phase 2, single ascending dose to multiple ascending dose)
				3a.3*	Statistical methods or rationale underpinning the trial design
				3a.4*	Starting dose(s) with rationale
				3a.5*	Range of planned dose levels with rationale
				3a.6*	Presentation of planned dose levels (eg, as a diagram, table, or infographic), where applicable
				3a.7*	Skipping of dose level(s), if applicable
				3a.8*	Planned cohort size(s) (eg, fixed, flexible, adaptive)
				3a.9*	Dose allocation method within a dose level (including sequence and interval between dosing of participants, eg, sentinel or staggered dosing)
				3a.10*	Dose expansion cohort(s), if applicable, with rationale
				3a.11*	Criteria for progression to the next part of the trial (eg, phase 1 to phase 2, single ascending dose to multiple ascending dose), where applicable
	3b	Important changes to methods after trial commencement (such as eligibility criteria), with reasons		3b†	Important changes to the design or methods after trial commencement (eg, insertion of unplanned additional doses) outside the scope of the prespecified adaptive design features, with reasons
Participants	4a	Eligibility criteria for participants		4a	
	4b	Settings and locations where the data were collected		4b	
Interventions	5	The interventions for each group with sufficient details to allow replication, including how and when they were actually administered		5a†	Interventions for each dose level (within each group) with sufficient details to allow replication, including administration route and schedule showing how and when they were actually administered
				5b*	Criteria for dose discontinuation, dose modifications, and dosing delays of allocated interventions for a given trial participant (eg, dose change in response to harms, participant request, or improving or worsening disease)
Outcomes	6a	Completely defined prespecified primary and secondary outcome measures, including how and when they were assessed		6a†	Primary and secondary outcomes, including the specific measurement variable, analysis metric, method of aggregation, and time point for each outcome. Explanation of the clinical relevance of chosen outcomes is strongly recommended. Any other outcomes used to inform prespecified adaptations should be described with the rationale
	6b	Any changes to trial outcomes after the trial commenced, with reasons		6b†	Any unplanned changes to trial outcomes after the trial commenced, with reasons
Sample size	7a	How sample size was determined		7a†	Estimated number of participants (minimum, maximum, or expected range) needed to address trial objectives and how it was determined, including clinical and statistical assumptions supporting any sample size and operating characteristics
	7b	When applicable, explanation of any interim analyses and stopping guidelines		7b†	Prespecified interim decision making criteria or rules that guided the trial adaptation process (eg, dosing decision to escalate or de-escalate); prespecified and actual timing and frequency of interim data reviews and the information to inform trial adaptations
**Randomisation (if applicable)**					
Sequence generation	8a	Method used to generate the random allocation sequence		8a	
	8b	Type of randomisation; details of any restriction (such as blocking and block size)		8b†	Type of randomisation; details of any restrictions (such as blocking and block size); any prespecified adaptive assignment rules or algorithm leading to adjustments in the allocation ratio, including timing and frequency of updates; any changes to the allocation rule following trial adaptation decisions
Allocation concealment mechanism	9	Mechanism used to implement the random allocation sequence (such as sequentially numbered containers), describing any steps taken to conceal the sequence until interventions were assigned		9	
Implementation	10	Who generated the random allocation sequence, who enrolled participants, and who assigned participants to interventions		10	
Blinding	11a	If done, who was blinded after assignment to interventions (eg, participants, care providers, and how		11a	
	11b	If relevant, description of the similarity of interventions		11b	
Statistical methods	12a	Statistical methods used to compare groups for primary and secondary outcomes		12a.1†	Statistical methods for primary and secondary outcomes and any other outcomes used to make prespecified adaptations
				12a.2*	For the implemented adaptive design features, statistical methods used for estimation (eg, safety, dose(s), treatment effects) and to make inferences
	12b	Methods for additional analyses, such as subgroup analyses and adjusted analyses		12b†	Statistical methods for additional analyses (eg, subgroup and adjusted analyses, pharmacokinetics or pharmacodynamics, biomarker correlative analyses)
				12c*	Analysis population(s) (eg, evaluable population for dose-finding, safety population)
				12d*	Strategies for handling intercurrent events occurring after treatment initiation (eg, how dosing adjustments were handled) that can affect either the interpretation or the existence of the measurements associated with the clinical question of interest, and any methods to handle missing data
**Results**					
Participant flow (a diagram is strongly recommended)	13a	For each group, the numbers of participants who were randomly assigned, received intended treatment, and were analysed for the primary outcome		13a†	For each group, the number of participants who were assigned to each dose level at each interim analysis (eg, for dosing decisions), received intended treatment, and were analysed for the primary outcome and, if applicable, any other outcomes used to inform prespecified adaptations
	13b	For each group, losses and exclusions after randomisation, together with reasons		13b†	For each group, losses and exclusions after allocation to each dose level, together with reasons
Recruitment	14a§	Dates defining the periods of recruitment and follow-up		14a§	
	14b§	Why the trial ended or was stopped		14b§	
				14c*	Trial adaptation decisions made (including on what basis they were made, and when) in light of the prespecified decision making criteria and observed accrued data
Baseline data	15	A table showing baseline demographic and clinical characteristics for each group		15†	Baseline demographic and clinical characteristics across each dose level within each group, where appropriate
Numbers analysed	16	For each group, number of participants (denominator) included in each analysis and whether the analysis was by original assigned groups		16†	For each group, the number of participants (denominator) included in each analysis across each dose level, and whether the analysis was by original assigned interventions
Outcomes and estimation	17a	For each primary and secondary outcome, results for each group, and the estimated effect size and its precision (such as 95% confidence interval)		17a†	For each primary and secondary outcome, results for each dose level within each group, and the estimated effect size and its precision, if applicable
	17b§	For binary outcomes, presentation of both absolute and relative effect sizes is recommended		17b§	
				17c*	Report interim results used to inform interim decision making such as dose escalation, de-escalation, or staying at the same dose
Ancillary analyses	18	Results of any other analyses performed, including subgroup analyses and adjusted analyses, distinguishing prespecified from exploratory		18	
Harms	19	All important harms or unintended effects in each group (for specific guidance, see CONSORT for harms^14^)		19†	All important harms (eg, adverse events or effects, toxicities) reported by dose level in each group (for specific guidance, see CONSORT for harms^52^)
**Discussion**					
Limitations	20	Trial limitations, addressing sources of potential bias, imprecision, and, if relevant, multiplicity of analyses		20	
Generalisability	21	Generalisability (external validity, applicability) of the trial findings		21	
Interpretation	22	Interpretation consistent with results, balancing benefits and harms, and considering other relevant evidence		22	
**Other information**					
Registration	23	Registration number and name of trial registry		23	
Protocol	24	Where the full trial protocol can be accessed, if available		24	
Funding	25	Sources of funding and other support (such as supply of drugs), role of funders		25	
Data monitoring				26a*	Composition of any decision making or safety review committee or group; summary of its role and reporting structure; statement of whether it is independent from the sponsor and competing interests; and reference to where further details can be found (such as in a charter or protocol)
				26b*	Description of who had access to interim results and made the interim and final decision to terminate the trial (or part(s) of the trial, eg, end of dose escalation), and measures to safeguard the confidentiality of interim information
Dissemination				27*	Specify, if applicable, whether and when results (such as safety and/or activity) were reported externally (eg, through scientific presentations, journal publication, or the trial website) while the trial (or part(s) of the trial) was still ongoing

CONSORT=CONsolidated Standards Of Reporting Trials; DEFINE=Dose-finding Extension; EPDF=early phase dose-finding. This checklist should be used in conjunction with the CONSORT explanation and elaboration document^37^ for important clarifications on the checklist items. Empty items in the CONSORT-DEFINE column indicate no modification from the standard CONSORT item. CONSORT extensions for non-pharmacological treatments and outcomes might also be relevant.^53^ Note that the term “dose” in the checklist can be considered synonymous and used interchangeably with dosage, or dosing regimen (dose or schedule), or a unit dose.

* New items that should only be applied in reference to CONSORT-DEFINE.

† Modified items that require reference to both CONSORT and CONSORT-DEFINE.

§ Item wording remains unchanged in reference to CONSORT, but additional CONSORT-DEFINE explanatory text has been provided to clarify additional considerations for early phase dose-finding trials (web appendix 3).

**Table 2:** CONSORT extension for abstracts and CONSORT-DEFINE for abstract extension checklists—items to include when reporting an early phase dose-finding trial in a journal or conference abstract

Section or item	CONSORT extension for abstracts	CONSORT-DEFINE for abstracts of EPDF trials
Title†	Identification as a randomised trial in the title	Identification as an early phase dose-finding (eg, dose escalation or de-escalation, phase 1, phase 1/2 or dose titration) trial in the title or abstract, and, if applicable, randomisation and/or trial acronym
Authors*	Contact details for the corresponding author	
Trial design†	Description of the trial design (eg, parallel, cluster, non-inferiority)	Description of trial design elements, such as dose escalation or de-escalation strategy, number of treatment groups, allocation ratio if relevant, and details of any prespecified trial adaptations
Methods		
Participants	Eligibility criteria for participants and the settings where the data were collected	
Interventions†	Interventions intended for each group	Interventions for each dose level within each group
Objective†	Specific objective or hypothesis	Specific objectives (eg, relating to safety, pharmacokinetics, pharmacodynamics, recommended dose(s))
Outcome	Clearly defined primary outcome for this report	Clearly defined primary outcome(s) for this report
Allocation	How participants were allocated to interventions	
Blinding (masking)	Whether or not participants, care givers, and those assessing the outcomes were blinded to group assignment	
Results		
Numbers allocated	Number of participants randomised to each group	Number of participants allocated to each group
Recruitment	Trial status	
Numbers analysed	Number of participants analysed in each group	
Outcome†	For the primary outcome, a result for each group and the estimated effect size and its precision	For the primary outcome(s), results for each dose within each group, and the estimated effect size and its precision, if applicable
Harms	Important adverse events or side effects	
Conclusions	General interpretation of the results	
Trial registration	Registration number and name of trial register	
Funding	Source of funding	

CONSORT=CONsolidated Standards Of Reporting Trials; DEFINE=Dose-finding Extension; EPDF=early phase dose-finding.

* This item is specific to conference abstracts.

† Modified items.

Variations in the terminology and definitions exist across disciplines and geographical areas in EPDF trials, so key terms used throughout this paper are provided in the glossary (box 1). We use CONSORT to refer to CONSORT 2010.

The CONSORT-DEFINE checklist includes several EPDF specific design items to provide a detailed elaboration of the trial design (eg, dosing strategies and adaptive features, dose allocation method, and expansion cohort(s)) to help readers understand dose adaptation strategies and other trial design adaptations. 

The specification of planned design adaptations and their scope are critical for preserving the integrity of adaptive designs and for regulatory assessments, as well as ensuring that the procedures and findings can be replicated.^18^ These factors have an impact on statistical methods for design and analysis; thus, CONSORT-DEFINE recommends providing comprehensive information on statistical methods that cover these adaptive features, as well as requiring clear definitions of analysis populations and plans for dealing with intercurrent events that occur after treatment initiation. 

Both analysis populations and intercurrent events are related to the estimands framework, which provides guidance on defining the treatment effect under investigation in a clinical trial (see the International Council for Harmonisation of Technical Requirements for Pharmaceuticals for Human Use (ICH) E9 (R1) addendum on estimands for further details^11^).^12^

As the results of key endpoints at each dose level are important findings of EPDF trials to understand the association between the dose of an intervention and its effects on participants, and to inform dose selection for subsequent trials, the CONSORT-DEFINE checklist also includes the additional requirement of reporting key results by dose level. This inclusion serves to enhance reproducibility, interpretation, usefulness of results, and evidence synthesis. 

For a detailed overview of new and modified checklist items specific to EPDF trials, see box 2.

**​Box 2: Overview of new and modified items in the CONSORT-DEFINE checklist**

**Title (one modified item)**

- Identification of features of EPDF trial in the title or abstract.

**Background (two new and two modified items)**

- Coverage of non-clinical or preclinical research informing an EPDF trial^36^ and any planned biomarker substudies^60^

**Methods (14 new and 10 modified items)**

- Elaboration of the trial design section to include dosing strategies and adaptive features,^18^ ^61^ range of planned dose levels, including starting dose(s) with rationale, dose allocation method (whether participants are dosed continuously or in a staggered way), and expansion cohort(s) where applicable with rationale^29^ ^36^ ^39^ ^57^ ^62^
- Enhanced intervention details,^5^ including prespecified criteria for dose discontinuation, dose modifications, or dose delays^36^
- Inclusion of clinical and statistical assumptions supporting the planned sample size and operating characteristics^25^ ^55^
- Specification of planned interim decision making criteria or rules and stopping guidelines to reflect the dosing decision process and other trial adaptations^18^ ^61^; progression criteria to move from one part of the trial to another where applicable (eg, from dose escalation to phase 2)^36^ ^57^
- Increased details regarding statistical methods to cover adaptive features, analysis populations, and intercurrent events that occur after treatment initiation^18^ ^55^

**Results (two new and six modified items)**

- Update of the results section to include reporting by dose levels^47^

**Data monitoring (two new items)**

- The addition of a new data monitoring section to cover decision making or safety review committees and descriptions of interim data reviews^18^

**Dissemination (one new item)**

- The addition of a new dissemination section to cover external reporting of ongoing trials

CONSORT=CONsolidated Standards Of Reporting Trials; DEFINE=Dose-finding Extension; EPDF=early phase dose-finding.

Minor wording changes were made to accommodate both randomised and non-randomised participant assignment; the term “where applicable” has been added to CONSORT 2010 items that might not apply to all EPDF trials. The wording of three CONSORT-DEFINE checklist items was elaborated to be consistent with the relevant items from the SPIRIT 2013 checklist.

Access to information relating to recommended items is most important. If not all the recommended information can be provided in the primary paper, authors can indicate where details can be found; for instance, in an accessible protocol, a statistical analysis plan, or a separate supplementary file. Authors should also provide explanations for items where details cannot be provided.

For items that remain unchanged, we refer users to the CONSORT 2010 explanation and elaboration document.^37^ The detailed explanation and rationale for the 40 new or modified CONSORT-DEFINE checklist items for the main report, along with examples from oncology and non-oncology settings, will be presented in a further publication by the authors. Here, we provide general comments and a brief overview of the items that might be less self-explanatory.

The CONSORT-DEFINE checklist recommends detailed elaboration of the trial design and statistical methods covering its adaptive features, including escalation or de-escalation strategies. CONSORT items 13a, 13b 15 16, and 17a were modified to add the requirement to report key results by dose level(s) or at each interim analysis for each intervention group or arm or specifically defined subgroups of interest (eg, healthy volunteers and patients, or young and older participants). Authors are encouraged to provide an explanation if the level of reporting might not be appropriate in certain settings, such as easily identifiable participants.

We recommend that authors provide a detailed description of the applicable statistical methods used to set up and implement the adaptive design in EPDF trials (item 3a.3). In the case of model based designs,^34^ it is important to explain the model assumptions, parameters, and mathematical form of the model. For both model based and model assisted dose-finding designs,^34^ ^54^ researchers should provide the rationale for choosing a target risk or toxicity rate or acceptable range,^33^ the dose transformation details (including the full skeleton and its elicitation), and bayesian prior distributions, if applicable.^55^ For rule based designs (such as 3+3, Rolling 6,^56^ single or multiple ascending dose^57^), the rationale for their use should be outlined. For other adaptations, such as early stopping for futility, research should clearly describe the statistical methods used (such as conditional power, predictive power, and posterior probability of treatment effect).^18^ ^55^

Authors should explain how they will deal with missing data and intercurrent events (item 12d), such as dosing delays, reductions, or interruptions, that occur after treatment initiation and could affect the interpretation or existence of measurements related to the clinical question.^11^ ^55^ These events might also include withdrawals of consent or unrelated deaths. Various strategies can be used to handle different types of missing data and intercurrent events, and sensitivity analyses can be conducted to assess the effect of the chosen strategies on trial results.^55^

The suggested abstract structure for EPDF trials, CONSORT-DEFINE for abstracts, follows a similar format as the 2008 CONSORT extension for journal and conference abstracts.^43^ The modifications are tailored to the objectives of EPDF trials (table 2). We outlined the recommended items that should be included in abstracts wherever possible. The level of detail could vary depending on the style and word count limit adopted by journals or conferences, as well as the complexity of the EPDF trial design. This extension should be used together with the CONSORT for journal and conference abstracts^43^ and any other relevant extensions. 

There are five modifications to the abstract guideline for EPDF trials. They affect the title (to highlight key features of EPDF trial to facilitate electronic searching); trial design (to provide a description of EPDF trial design); methods (to specify doses used and objectives of EPDF trials, such as safety and recommended doses); and results (to include the provision of primary outcomes at each dose (where possible) in each group). Other minor refinements include the term “randomised” being replaced with “allocated,” to account for randomised or non-randomised EPDF trials, and the allowance of one or joint primary outcomes, which is not an uncommon feature in EPDF trials.

## Discussion

CONSORT-DEFINE provides international evidence and consensus based guidance for reporting EPDF trials in main reports and journal or conference abstracts. It extends the CONSORT checklist for the main trial report by introducing 40 items. These include 21 new items and 19 modifications to existing CONSORT items tailored specifically to EPDF trials. CONSORT-DEFINE is designed to be used alongside the latest CONSORT guidance. Additionally, CONSORT-DEFINE includes five modifications to the existing CONSORT abstract recommendations^43^ for improved reporting of EPDF trial abstracts.

CONSORT-DEFINE, like other CONSORT extensions, was developed through an international consensus driven process using the EQUATOR methodological framework. Its unique focus lies in addressing the distinctive features of EPDF trials.

We also developed a dose-finding extension for SPIRIT 2013,^46^ SPIRIT-DEFINE, which has been reported elsewhere.^45^ CONSORT-DEFINE and SPIRIT-DEFINE together form an interconnected continuum designed to facilitate the writing of the trial protocol and report, as well as assess the adherence of the final report to the protocol.^58^

### Application of CONSORT-DEFINE

The CONSORT-DEFINE guidance aims to be a useful resource for trialists, journal editors, peer reviewers, funders, regulators, and research ethics committees to promote best practice in reporting EPDF trials. We also envision that it will enable both trial participants and the public to be more confident in EPDF trial design and results. 

CONSORT-DEFINE presents the minimum essential items that should be reported for EPDF trials to maximise their transparency, replication, and usefulness and limit selective reporting of their results. There will, however, be settings where reporting additional items might be viewed as important, especially for complex trial designs. Like CONSORT, CONSORT-DEFINE guidance is not prescriptive regarding the structure or location of the required information; authors are encouraged to “address checklist items somewhere in the article, with ample detail and lucidity,”^38^ or to indicate where details can be found (eg, in an accessible protocol, statistical analysis plan, or supplementary documents).

When applying the CONSORT-DEFINE guidance, authors can indicate why any recommended item might not be applicable to their trial. For instance, providing key findings from relevant non-clinical or preclinical research (item 2a.2) might be deemed unnecessary in paediatric trials of drugs for which there is ample evidence of usage in adults, especially when the disease is the same or very similar in adults and children^59^ (covered in item 2a.1).

It is important to note that CONSORT-DEFINE is a reporting guideline and is thus not intended to advocate for any specific trial design. Its focus is to provide the minimum essential contents for transparent and complete reporting of the design, conduct, analyses, and interpretation of the conducted early phase dose-finding clinical trial (including what was planned and what was actually implemented), regardless of the trial design used, to enable readers to critically and comprehensively assess the validity and reliability of the trial results.

### Key strengths and limitations

The development of this checklist extension had noteworthy strengths and limitations. We successfully engaged more than 200 multidisciplinary stakeholders from 24 countries in the Delphi survey to develop these guidelines and promote international awareness and usability. However, the survey results are not immune to non-response bias. Participants were self-selecting, because only those interested would sign up to participate in the Delphi survey, and we were unable to determine the experiences or characteristics of those who did not opt to participate.

Moreover, throughout the development process, we successfully engaged with patients and public partners. Apart from the participation of two patient representatives at the DEFINE consensus meeting, who played an important part in shaping the eventual checklist, our PPIE efforts also resulted in the coproduction of a toolkit for creating lay summaries of EPDF trial reports.^48^ Such engagement in the development of reporting guidance has been rare to date and should be strongly encouraged to ensure that patients’ voices are taken into account.

Around 16% of registered participants did not complete their round one survey despite at least three reminders. This non-completion could be due to the length of the survey (80 questions for both SPIRIT-DEFINE and CONSORT-DEFINE), which would have required around 30 minutes to complete. To mitigate this, we tried to reduce the time taken by displaying each new or modified item that is relevant to both the SPIRIT-DEFINE and CONSORT-DEFINE candidate items at the same time in the survey to reduce participant fatigue; the save functionality also permitted the survey to be completed in multiple sessions.

The consensus participants were purposefully chosen from commercial and non-commercial organisations, including PPIE representatives, to reflect different expertise and job roles relevant to trial design, conduct, and reporting. However, some groups that were less well represented in the consensus meeting panel (eg, those outside Europe, North America, and Asia) might have different views. Nonetheless, the systematic and evidence based approach used to develop this guideline, which included a comprehensive review of reporting practices on EPDF trials and the widespread engagement in the Delphi survey, might have mitigated the potential effect of these limitations.^2^ ^44^

CONSORT-DFEINE is generic to cover diverse trial designs that are applicable in EPDF trials. The design of an EPDF trial is generally more multifaceted than that of a two arm, parallel group trial. A major strength of the CONSORT-DEFINE guidance is that, while based on the original CONSORT, it has also been refined to reflect the distinctive purposes and characteristics of EPDF clinical trials through a specific extension. These characteristics can include diverse populations (healthy volunteers or patients), interventions (pharmacological, non-pharmacological, or a combination of both), and trial designs that might range from pharmacokinetic modelling in healthy volunteers to complex bayesian modelling of joint outcomes such as toxicity and activity. Consequently, researchers could find some new or modified items in this extension difficult to adhere to. We, therefore, intentionally kept some items separate as individual items rather than combining them as a composite item to ensure that they would not be missed in reporting. For instance, CONSORT-DEFINE 2a.1, 2a.2, and 2a.3 were kept separate rather than combined as one modified item of CONSORT 2a. Similarly, for the trial design, CONSORT-DEFINE 3a.1-3a.11 were kept separate as 11 individual items rather than as a composite modified item of CONSORT item 3a.

### Enhancing the uptake and relevance of CONSORT-DEFINE

Wide dissemination of the CONSORT-DEFINE guidance is essential to increasing its appropriate uptake, and this will be done as previously outlined,^2^ ^29^ including to journals currently known to endorse CONSORT through the EQUATOR Network. Additionally, we are preparing an explanation and elaboration document to provide in-depth details and exemplars from published papers in different settings, to assist reviewers, editors, and readers who require additional information or clarity about specific items.

The landscape of EPDF trial design is rapidly evolving, with an increasing use of seamless phases as well as innovative and efficient approaches to fulfil multiple objectives with faster decisions. Additional considerations could be needed as newer trial designs emerge. The DEFINE executive committee will monitor the literature and assess the need to update the CONSORT-DEFINE guidance accordingly. Users are encouraged to provide any feedback on the content, usability, and clarity of the guidance and how it can be further refined, which will be used to shape future updates.

### Conclusions

This robust, consensus driven CONSORT-DEFINE guidance empowers researchers to effectively address the essential items that should be included in EPDF trial reports. In doing so, it promotes greater transparency, reproducibility, informativeness, and usefulness of results, which in turn will enhance the trustworthiness of EPDF trials with patients and the public.

## Web extra

Extra material supplied by authors

Web appendix 1: Supplementary materials

Web appendix 2: CONSORT-DEFINE downloadable checklist

Web appendix 3: Clarification for the items that remained unchanged with reference to CONSORT

Web appendix 4: Acknowledgment of contributors to the development of CONSORT-DEFINE guidance

## Notes

Contributors: MD and CJW contributed equally. CY, OS, JdB, TJ, AM, TRJE, SH, KRR, MC, SL, AK, A-WC, AE, MD, and CJW conceived the study. CY, OS, JR, and DP curated the data. JR and DP conducted the formal analysis. CY, JdB, TJ, AM, TRJE, SH, SL, AK, MD, and CJW acquired the funding. CY, OS, JdB, TJ, AM, TRJE, SH, MU, KRR, MC, SL, AK, AE, MD, and CJW contributed to the methodology. CY, OS, DP, and AE conducted the project administration. CY, SH, MC, AK, DA, A-WC, EG-M, JDI, MD, and CJW supervised the project. CY, OS, JdB, JR, TJ, RP, KSH, MU, MC, MD, and CJW wrote the original draft. All authors were involved in the investigation; and in the reviewing and editing of the manuscript. CY is the guarantor. The corresponding author attests that all listed authors meet authorship criteria and that no others meeting the criteria have been omitted.

Patient and public involvement: The DEFINE study patient and public involvement engagement (PPIE) lead (AK) has contributed to the design of the study and the development of the protocol since the early stages. We also sought out additional PPIE representatives from both the oncology and non-oncology fields to review the checklists to ensure that the view of patients and participants was adequately represented. In collaboration with these representatives, we developed a toolkit for lay reporting of early phase trial results,^48^ and we plan to produce lay publications to chart the development of both the SPIRIT-DEFINE and CONSORT-DEFINE guidelines.

Provenance and peer review: Not commissioned; externally peer reviewed.
