# Supplementary material for: Enhancing reporting quality and impact of early phase dose-finding clinical trials: CONSORT Dose-finding Extension (CONSORT-DEFINE) guidance
Source: BMJ. 2023 Oct 20;383:e076387. doi: 10.1136/bmj-2023-076387 (PMC10583500; doi:10.1136/bmj-2023-076387)
Supplement: Supplementary file 1 — Web appendix 1: Supplementary materials [file yapc076387.ww1.pdf]

Web appendix 1: Supplementary materials

Figure S1. Flow of CONSORT-DEFINE candidate items through the Delphi survey and DEFINE Consensus Meeting, including the decision criteria for inclusion in the checklist and the process for handling items that did not reach consensus

Table S1. CONSORT-DEFINE candidate items discussed at the DEFINE Consensus Meeting and voting results

Table S2. List of DEFINE International Consensus Meeting participants

Table S3. DEFINE Consensus Meeting participant characteristics

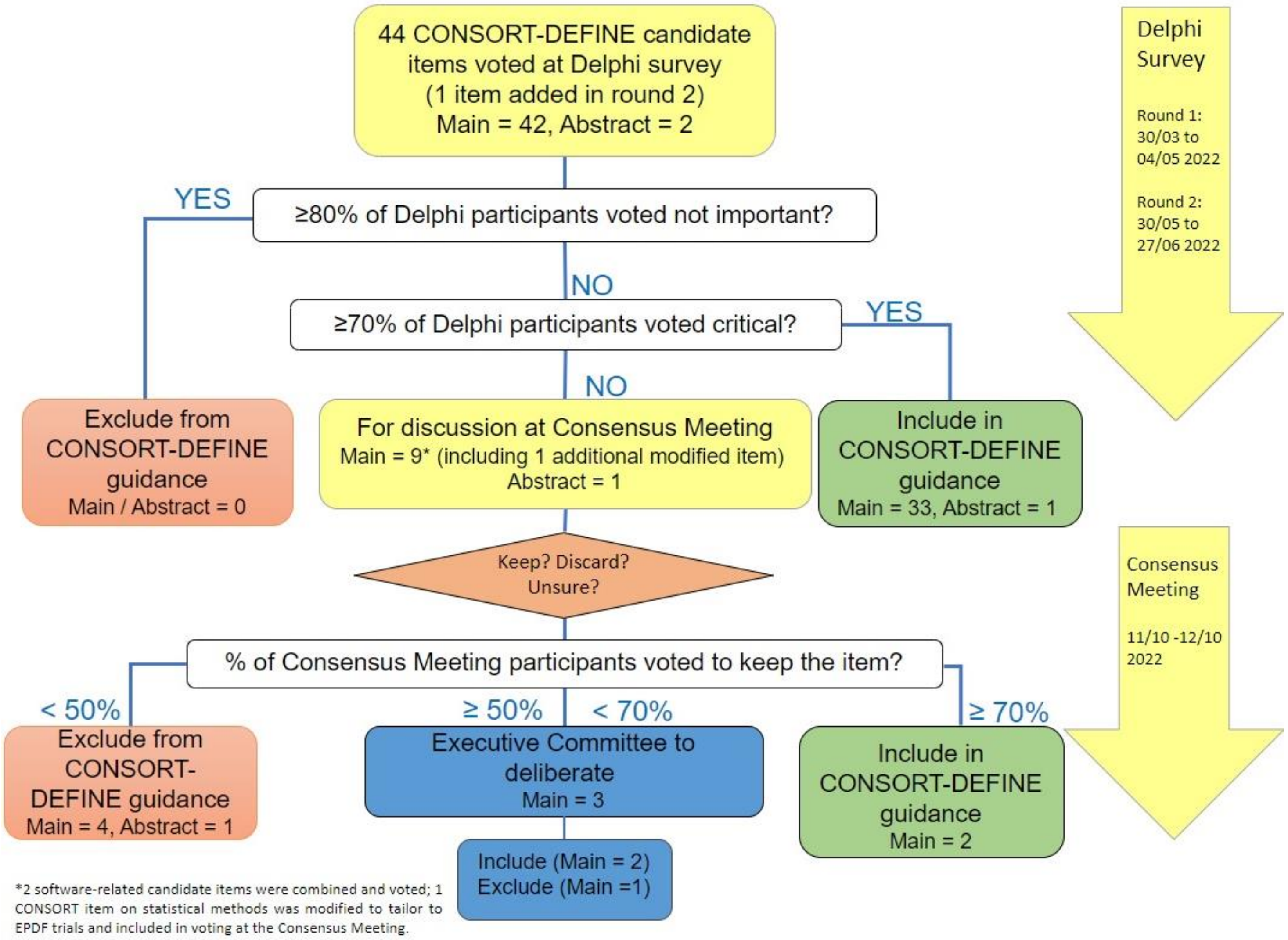

Figure S1. Flow of CONSORT-DEFINE candidate items through the Delphi survey and DEFINE Consensus Meeting, including the decision criteria for including the items in the checklist and the process for handling items that did not reach consensus

Table S1. CONSORT-DEFINE candidate items discussed at the DEFINE Consensus Meeting and voting results

CONSORT-DEFINE candidate items discussed at the DEFINE Consensus Meeting			DEFINE Consensus Meeting				Executive Committee decision
Topic	Delphi item number	Delphi Item description	Voting x/n (%)			Decision	
			Keep	Discard	Unsure		
Statistical methods (ACE-related item)	*Not in Delphi	Statistical methods used for primary and secondary outcomes, and **any other outcomes used to make pre-planned adaptations**	25/26 (96)	1/26 (4)	0/26 (0)	KEEP	N/A
Reporting of on-going trial results	69N	Specify if and when results (e.g., safety/response outcomes) were reported whilst the trial was still ongoing	25/30 (83)	5/30 (17)	0/30 (0)	KEEP	N/A
Biomarkers	6N	Summary of findings from existing correlative biomarker, correlative and associated studies to support planned biomarker sub-study (if applicable)	14/24 (58)	10/24 (42)	0/24 (0)	UNSURE	KEEP
Dosing regimens	18N	Planned and delivered dosing regimens presented as a diagram or table, where applicable	13/26 (50)	11/26 (42)	2/26 (8)	UNSURE	KEEP
Statistical software (combined w/ access link for simulations)	*55N (combined with 80N)	Statistical software and packages used for design (e.g., with access, or link to code/functions used for simulation studies) and analyses	13/26 (50)	9/26 (35)	4/26 (15)	UNSURE	DISCARD
Other relevant documents	72N	Where the full statistical analysis plan and other relevant trial documents (Oversight Committee, Safety Review/Data Monitoring Committee Charter, quality aspects of investigational medicinal product, investigators brochure, simulation report, this list is non-exhaustive) can be accessed	4/25 (16)	18/25 (72)	3/25 (12)	DISCARD	N/A
PPIE	75N	Involvement of patients, service users, their carers, members of public or patient advocates in any aspect of the trial and or reason why their involvement is not necessary	10/26 (38)	15/26 (58)	1/26 (4)	DISCARD	N/A
Lay summary	77N	Lay summary of the trial results or where it can be accessed	7/25 (28)	17/25 (68)	1/25 (4)	DISCARD	N/A

CONSORT-DEFINE candidate items discussed at the DEFINE Consensus Meeting			DEFINE Consensus Meeting				Executive Committee decision
Topic	Delphi item number	Delphi Item description	Voting x/n (%)			Decision	
			Keep	Discard	Unsure		
Access to full protocol/redacted version	70M	Where the full trial protocol or the redacted version, with amendments (if any), can be accessed	7/29 (24)	21/29 (72)	1/29 (3)	DISCARD	N/A
Abstract - adaptations made	79N	Dose decisions/adaptations were made in light of pre-planned decision-making criteria and observed accrued data	8/28 (29)	19/28 (68)	1/28 (4)	DISCARD	N/A

Two items were voted to be included and five were excluded from the CONSORT-DEFINE checklist at the DEFINE Consensus Meeting. Three candidate items that did not reach consensus were deliberated at the Executive Committee meeting, with the final decision to include two items in the checklist and exclude one.

N = New item; M = Modified item

* 2 software-related candidate items were combined and voted; 1 CONSORT item on statistical methods was modified to tailor to EPDF trials and included in voting at the Consensus Meeting.

Table S2. List of DEFINE International Consensus Meeting participants

Name [a]	Affiliation(s)	Country of work	Self-reported Stakeholder Group
1. Adrian Mander	GlaxoSmithKline	United Kingdom	Statistician/Trial methodologist
2. Agnes V Klein	Health Canada	Canada	Clinician;<br>Regulator
3. Andrew Kightley	Tarsius	United Kingdom	Patient and Participant Involvement representative
4. Antoine Hommais	Institut National du Cancer,	France	Funder
5. An-Wen Chan	University of Toronto,	Canada	Clinician;<br>Ethics;<br>Journal editor;<br>Statistician/Trial methodologist
6. Christina Yap<br>(Principal Investigator)	The Institute of Cancer Research	United Kingdom	Funder;<br>Journal editor;<br>Regulator;<br>Statistician/Trial methodologist
7. Christopher J Weir	University of Edinburgh	United Kingdom	Regulator;<br>Statistician/Trial methodologist
8. Dawn Richards	Clinical Trials Ontario	Canada	Patient and Participant Involvement representative
9. Deborah Ashby<br>(Chair of DEFINE Consensus Meeting)	Imperial College London	United Kingdom	Statistician/Trial methodologist
10. Elizabeth Garrett-Mayer	American Society of Clinical Oncology	United States of America	Ethics;<br>Journal editor;<br>Statistician/Trial methodologist
11. James Matcham	Cytel	Australia	Statistician/Trial methodologist
12. Thomas R Jeffry Evans	University of Glasgow	United Kingdom	Clinician;<br>Journal editor
13. Johann de Bono	The Institute of Cancer Research<br><br>The Royal Marsden NHS Foundation Trust	United Kingdom	Clinician
14. John Isaacs	Newcastle University	United Kingdom	Clinician;<br>Funder;<br>Journal editor
15. Jordan Berlin	Vanderbilt-Ingram Cancer Center	United States of America	Clinician
16. Kathryn S Hayward	University of Melbourne	Australia	Clinician;<br>Trial management
17. Kate Williams [c]	Health Research Authority	United Kingdom	Clinician;<br>Ethics;
18. Khadija Rerhou Rantell	Medicines and Healthcare products Regulatory Agency	United Kingdom	Statistician/Trial methodologist
19. Lesley McGuigan	Cancer Research UK	United Kingdom	Trial Management
20. Lesley Seymour	Queens University	Canada	Clinician;<br>Trial management

Name [a]	Affiliation(s)	Country of work	Self-reported Stakeholder Group
			Statistician/Trial methodologist
21. Lynley V Marshall	Institute of Cancer Research<br><br>The Royal Marsden Hospital NHS Foundation Trust	United Kingdom	Clinician
22. Melanie Calvert	Birmingham University	United Kingdom	Statistician/Trial methodologist
23. Mirat Shah [b]	Food and Drug Administration	United States of America	
24. Moreno Ursino	INSERM – French National Health Institute	France	Statistician/Trial methodologist
25. Munyaradzi Dimairo	University of Sheffield	United Kingdom	Funder;<br>Journal editor;<br>Statistician/Trial methodologist
26. Olga Kholmanskikh	European Medicines Agency	Belgium	Regulator
27. Oliver Boix	Bayer	Germany	Statisticians/Trial methodologist
28. Paul Frankel	Journal of Clinical Oncology<br><br>City of Hope Comprehensive Cancer Centre	United States of America	Ethics;<br>Journal editor;<br>Statistician/Trial methodologist
29. Richard Peck	University of Liverpool<br>F Hoffmann la Roche	France	Clinician
30. Robert Golub	Journal of the American Medical Association	United States of America	Journal editor
31. Rong Liu	Bristol-Myers Squibb	United States of America	Statistician/Trial methodologist
32. Sally Hopewell	University of Oxford	United Kingdom	Statistician/Trial methodologist
33. Shing Lee	Columbia University	United States of America	Statistician/Trial methodologist
34. Susan Percy Ivy	National Institute of Health	United States of America	Other (Regulatory Medical Officer)
35. Yoshiya Tanaka	University of Occupational and Environmental Health	Japan	Clinician;<br>Journal editor

[a] DEFINE team members who helped facilitate the meeting but did not vote or take part in discussions: Aude Espinasse, Dhrusti Patel, Jan Rekowski, and Olga Solovyeva.
[b] Mirat Shah took part in the consensus meeting discussion but did not vote on any items.
[c] Kate Williams did not attend the consensus meeting or vote for any items but provided feedback via email.

Table S3. DEFINE Consensus Meeting participant characteristics

Participant Characteristics	n (%)
**Roles** *(multiple entries were permitted)*	
Clinician / clinical pharmacologist	12 (34.3)
Trial management staff (including trial manager / coordinator and data manager)	3 (8.6)
Statistician / trial methodologist / data scientist / quantitative analyst	17 (48.6)
Regulator / Regulatory assessor or advisor	6 (17.1)
Ethics committee / institutional review board member	4 (11.4)
Journal editor, associate editor, and conference abstracts committee member	9 (25.7)
Funder / funding committee member	4 (11.4)
Patient and Public Involvement representative / contributor / Patient Advocate	2 (5.7)
Other:	2 (5.7)
**Number of roles**	
1	21 (60.0)
2 or more	14 (40.0)
**Clinical trials research experience (years)**	
< 1	1 (2.9)
1-5	2 (5.7)
6-14	7 (20.0)
15 or more	25 (71.4)
**Early phase clinical trials experience (years)**	
< 1	2 (5.7)
1-5	5 (14.3)
6-14	7 (20.0)
15 or more	21 (60.0)
**Type of clinical trials experience**	
Cancer patient trials	9 (25.7)
Non-cancer patient trials	5 (14.3)
Cancer patient trials + non-cancer patient trials	5 (14.3)
Trials involving healthy volunteers + non-cancer patient trials	1 (2.9)
All of the above	10 (28.6)
None of the above	5 (14.3)
**Country**	
Australia	2 (5.7)
Belgium	1 (2.9)
Canada	4 (11.4)
France	3 (8.6)
Germany	1 (2.9)
Japan	1 (2.9)
United Kingdom	15 (42.9)
United States of America	8 (22.9)
