# Supplementary material for: Enhancing reporting quality and impact of early phase dose-finding clinical trials: CONSORT Dose-finding Extension (CONSORT-DEFINE) guidance
Source: BMJ. 2023 Oct 20;383:e076387. doi: 10.1136/bmj-2023-076387 (PMC10583500; doi:10.1136/bmj-2023-076387)
Supplement: Supplementary file 3 — Web appendix 3: Clarification for the items that remained unchanged with reference to CONSORT [file yapc076387.ww3.pdf]

Web appendix 3: Clarification for the items that remained unchanged with reference to CONSORT

Three items have remained unchanged with reference to CONSORT, but additional explanatory text has been provided for further clarity regarding considerations for early phase dose-finding trials.

Item 14a§ Dates defining the periods of recruitment and follow-up

In the case of trials with different parts or major transition points (e.g., seamless trials, escalation/expansion, phase I/II, SAD/MAD), it would be useful to provide separate periods of recruitment and follow-up.

Item 14b§ Why the trial ended or was stopped

This item will cover why the trial ended or was stopped outside the scope of pre-specified adaptations (e.g., due to poor recruitment or withdrawal of trial funding). The standard CONSORT item does not differentiate between planned and unplanned criteria for stopping early. Stopping the trial early due to pre-specified adaptations is covered in item 14c.

Item 17b§ For binary outcomes, presentation of both absolute and relative effect sizes is recommended

This item might only apply in specific circumstances, for instance, in trials with larger sample sizes. For details, see CONSORT 2010 Explanation and Elaboration (1).

Reference:

1. Moher D, Hopewell S, Schulz KF, Montori V, Gotzsche PC, Devereaux PJ, et al. CONSORT 2010 Explanation and Elaboration: Updated guidelines for reporting parallel group randomised trials. J Clin Epidemiol. 2010;63(8):e1-37.
