# Supplementary material for: Enhancing reporting quality and impact of early phase dose-finding clinical trials: CONSORT Dose-finding Extension (CONSORT-DEFINE) guidance
Source: BMJ. 2023 Oct 20;383:e076387. doi: 10.1136/bmj-2023-076387 (PMC10583500; doi:10.1136/bmj-2023-076387)
Supplement: Supplementary file 4 — Web appendix 4: Acknowledgment of contributors to the development of CONSORT-DEFINE guidance [file yapc076387.ww4.pdf]

Web appendix 4: Acknowledgment of contributors to the development of CONSORT-DEFINE guidance

Research Team: Christina Yap (Principal Investigator), Olga Solovyeva (CONSORT-DEFINE lead methodologist), Jan Rekowski (SPIRIT-DEFINE lead methodologist), Dhrusti Patel (Research assistant), Aude Espinasse (Project manager).

Executive Committee and Collaborators/Advisors: Christina Yap (Principal Investigator), Institute of Cancer Research, United Kingdom; Munyaradzi Dimairo, University of Sheffield, United Kingdom; Christopher Weir, University of Edinburgh, United Kingdom; Adrian Mander , GlaxoSmithKline, United Kingdom; Thomas Jaki, University of Cambridge, United Kingdom; Jeff Evans, University of Glasgow, United Kingdom; Rong Liu, Bristol-Myers Squibb, US ; Shing Lee, Columbia University, US; Andrew Kightley (DEFINE PPIE Lead), Tarsius, United Kingdom; Sally Hopewell, University of Oxford, United Kingdom; Johann de Bono, Institute of Cancer Research, United Kingdom; Alun Bedding, Roche, United Kingdom; Stephen Hahn, Flagship Pioneering, US; Khadija Rantell , Medicines and Healthcare products Regulatory Agency, United Kingdom.

Independent Expert Panel: Elizabeth Garret-Mayer (chair), American Society of Clinical Oncology, US; Deborah Ashby, Imperial College London, United Kingdom; John Isaacs, Newcastle University, United Kingdom; Melanie Calvert, Birmingham University, United Kingdom.

Users who pilot-tested the CONSORT-DEFINE checklist: Kathryn S. Hayward, University of Melbourne, Australia; Olga Kholmanskikh, Federal Agency for Medicines and Health Products, Belgium; Christina Guo, The Institute of Cancer Research, United Kingdom; Courtney Coschi, Canadian Cancer Trials Group, Canada; Sian Lax, Cancer Research United Kingdom Clinical Trials Unit, University of Birmingham, United Kingdom.

DEFINE Delphi participants who consented to be acknowledged:

Agnes V Klein, Canada; Aidan Hindley, United Kingdom; Akshay A Shivchhand, India; Alastair Greystoke, United Kingdom; Alessandro Matano, Switzerland; Alexander Ooms, United Kingdom; Alun Bedding, United Kingdom; Andrew Althouse, United States of America; Andrew JT George, United Kingdom; Andy Vail, United Kingdom; Anna Zachariou, United Kingdom; Anne Loeser, United States of America; Annette Kopp-Schneider, Germany; Anthony Joshua, Australia; Aude Espinasse, United Kingdom; Becky M Salisbury, United Kingdom; Benjamin Johnson, United Kingdom; Björn Holzhauer, Switzerland; Carine Bellera, France; Caroline Kelly, United Kingdom; Catey Bunce, United Kingdom; Catherine Fullwood, United Kingdom; Christian Dittrich, Austria; Christian Gluud, Denmark; Christin Henein, United Kingdom; Christophe Le Tourneau, France; Christophe Massard, France; Clare Peckitt, United Kingdom; Conrad Fernandez, Canada; Crescens Tiu, United Kingdom; David Murray, United Kingdom; Dawn Richards, Canada; Deborah Ashby, United Kingdom; Debra J Lett, United Kingdom; Diana R Elbourne, United Kingdom; Elizabeth Garrett-Mayer, United

States of America; Elli Bourmpaki, United Kingdom; Elspeth Banks, United Kingdom; Emily Dressler, United States of America; Emily Greenlay, United Kingdom; Eric Frison, France; Fangrong Yan, China; Fiona Thistlethwaite, United Kingdom; Fred Cohen, United States of America; Gaëlle Saint-Hilary, France; Geert Jan Jan Groeneveld, Netherlands; Haitao Pan, United States of America; Helen Brittain, United Kingdom; Hemant Arora, United Kingdom; Herbert H. Loong, China; Ileana Baldi, Italy; Jan Rekowski, United Kingdom; Jean-Francois Pittet, United States of America; Jenna Grabey, United Kingdom; Joanna Moschandreas, United Kingdom; Johann de Bono, United Kingdom; John D Isaacs, United Kingdom; Jörg Haier, Germany; Joseph Ross, United States of America; Joshua Savage, United Kingdom; Jost B. Jonas, Germany; Karim M Khan, Canada; Kate Hayward, Australia; Kathrine J Craig, United Kingdom; Katrina Walker, United Kingdom; Kentaro Takeda, United States of America; Kosuke Kashiwabara, Japan; Kyun-Seop Bae, South Korea; Laura Richert, France; Lauren Walker, United Kingdom; Lesley K Seymour, Canada; Lewis L. Hsu, United States of America; Lisa Belin, France; Liz-Anne Lewsley, United Kingdom; Lukas Widmer, Switzerland; Lynley Marshall, United Kingdom; Marcio Augusto Diniz, United States of America; Maria Beatrice Panico, United Kingdom; Marina Pulido, France; Matthew Schipper, United States of America; Matthew Sydes, United Kingdom; Melanie Calvert, United Kingdom; Michael Grayling, United Kingdom; Muhammad Irfan bin Abdul Jalal, Malaysia; Nandi Siegfried, South Africa; Nuria Kotecki, Belgium; Olga Kholmanskikh, Belgium; Olga Solovyeva, United Kingdom; Oliver Boix, Germany; Paul Bycott, United States of America; Pedro A. Torres-Saavedra, United States of America; Peter Dewland, United Kingdom; Peter J Gill, Canada; Philip Drennan, United Kingdom; Rebecca Kristeleit, United Kingdom; Robert M. Golub, United States of America; Roger Dmochowski, United States of America; Rongji Mu, China; Ruitao Lin, United States of America; Sebastian Bate, United Kingdom; Sharon Love, United Kingdom; Siew Wan Hee, United Kingdom; Simon Crabb, United Kingdom; Simon Pacey, United Kingdom; Sonia Fox, United Kingdom; Stefan N. Symeonides, United Kingdom; Stephen Senn, United Kingdom; Susan E. Bates, United States of America; Susan Percy Ivy, United States of America; Susan Smith, United Kingdom; Sze Huey Tan, Singapore; Temsunaro Rongsen Chandola, India; Thomas J. Prior, United States of America; Tom Parke, United Kingdom; Victoria Homer, United Kingdom; Vivianne Shih, Singapore; Yoshiya Tanaka, Japan; Yung-Jue Bang, South Korea.

Other acknowledgements:

The authors thank the late Professor Doug Altman for his enthusiasm, inspiration, and significant contribution to the initial conception of this work; Siew Wan Hee, Sarah Hughes, Stephen Hahn, John Kirkpatrick, Alun Bedding, Kate Williams, and DEFINE PPIE working group (1), for their involvement in the early development stages of the DEFINE guidance, and Claire Snowdon for proofreading the manuscript.

References

1. Solovyeva O, Dimairo M, Weir CJ, Hee SW, Espinasse A, Ursino M, et al. Development of consensus-driven SPIRIT and CONSORT extensions for early phase dose-finding trials: the DEFINE study. BMC Med. 2023;21(1):246.
